# “The cord is the child”: meanings and practices related to umbilical cord care in Central Uganda

**DOI:** 10.1186/s12887-020-2002-9

**Published:** 2020-03-04

**Authors:** David Mukunya, Marte E. S. Haaland, James K. Tumwine, Thorkild Tylleskar, Victoria Nankabirwa, Karen Marie Moland

**Affiliations:** 1 Centre for Intervention Science in Maternal and Child Health (www.cismac.org), Center for International Health, University of Bergen, Bergen, Norway; 2 CISMAC, Center for International Health, University of Bergen, Bergen, Norway; 3 Department of Pediatrics and Child Health, Makerere University, Kampala, Uganda; 4 Department of Epidemiology and Biostatistics, School of Public Health, Makerere University, Kampala, Uganda

**Keywords:** Umbilical cord, Newborn care, Neonatal care, Sepsis, Chlorhexidine

## Abstract

**Background:**

Infections account for a quarter of all newborn deaths and the umbilical cord has been identified as a major route of newborn infections.

**Objective:**

To explore the meanings and practices related to the umbilical cord among caretakers of newborns in central Uganda.

**Methods:**

This was a qualitative study, designed to inform the design, and interpretation of a randomized controlled trial assessing the effectiveness of chlorhexidine use for the umbilical cord. We conducted 22 in-depth interviews exploring umbilical cord care practices among ten mothers, four health workers, five traditional birth attendants, and three men. We also conducted three focus group discussions with young mothers and elderly women. We used qualitative content analysis to analyze our findings and we borrow upon Mary Douglas’ concepts of dirt to present our findings.

**Results:**

The umbilical cord had a symbolic position in newborn care. The way it was perceived and handled had far reaching consequences for the survival and wellbeing of the baby. The umbilical cord was a centre of anxiety, a possible gate to illness, a test of fatherhood and a signifier of parental responsibility. Hence, the umbilical cord and the way it was cared for played a part in the present and future survival of the baby, as well as the survival and wellbeing of the household. Persons other than the mother such as older female relatives were very influential in the care of the umbilical cord.

**Conclusions:**

The umbilical cord carried symbolic meanings, which extended beyond the newborn and the newborn period, and in turn influenced the various practices of umbilical cord care. The important position of the cord in local newborn care practices should be recognized and taken into consideration when scaling up newborn care interventions in the country.

## Background

In sub-Saharan Africa, umbilical cord care is embedded in larger cultural constructs such as rites of passage [1], and fertility [2]. Substances such as salty water, soot, banana ash, herbs, surgical spirit, powder, ghee, papyrus reeds, saliva, water, butter and petroleum jelly are commonly applied to the cord [3–5], but vary with the region and cultural group [6]. These cultural elaborations [6, 7] and beliefs associated with the umbilical cord may increase the risk of umbilical cord infections [8–10], which often progress to neonatal sepsis. Neonatal sepsis accounts for almost a quarter of all newborn deaths [11] and contributes significantly to deaths from other causes such as prematurity [12]. Neonatal sepsis is also associated with neurodevelopment complications like cerebral palsy [13, 14] and impaired motor development [15]. As a result, interventions that promote hygienic umbilical cord care are listed as prioritized interventions in newborn health [12, 16]. Understanding umbilical cord care practices and the rationale behind these practices is vital for the scale up of interventions addressing umbilical cord care [2, 6], but very few studies have addressed this issue [2].

Therefore, in this paper, we explore practices associated with umbilical cord care in Central Uganda, and the meanings attached to the cord in this social and cultural context. To aid in the interpretation of our findings, we draw upon Mary Douglas’ concept of dirt as elaborated in her classical book in social anthropology *Purity and Danger* (1966) [17], and the concept of risk as elaborated in her later book *Risk and Blame* (1992) [18]. Mary Douglas aims to enhance our understanding of ideas and rituals related to cleanliness and pollution [17]. Dirt, defined as ‘matter out of place’, is seen as a contextual rather than an intrinsic characteristic of an item. Mary Douglas argues that we call something dirty because it defies the order or normal separateness of various states [17]. Likewise she sees risk as culturally constructed rather than an individual’s cognitive assessment [18], and she argues that it is closely linked to blame. People do not make decisions involving risk without consulting others lest they may get blamed if something goes wrong. These ideas are as relevant to the analysis of human practices and meaning making today and are extensively used in the literature on risk and in the analysis of bodily processes [19]. However, to our knowledge, her insights have not been much used in the analysis of newborn care. As we elaborate in this paper, the umbilical cord is filled with meanings related to both purity and to risk or danger, carrying with it hopes for the baby’s health and survival, and fear of blame for illness and misfortune. Mary Douglas is concerned with social and bodily boundaries and states that when something does not fit into one bounded category, anxiety is created. One way of dealing with this anxiety is eliminating the item [20], in our case the umbilical cord. We will use this insight in our analysis of the transition of the baby from inside the mother’s womb to the outside, and how this informs the meaning and practices related to the umbilical cord.

## Methods

The study was conducted between June 2016 and January 2017 in Mukono district in Central Uganda in a village neighbouring the health centre, an area where most people live as subsistence farmers and are largely stationary. Mukono district is approximately 20 km east of the capital city Kampala and has a population of approximately 600,000 people of which 340,000 are below the age of 18 years. Trade and farming are the most common economic activities while fishing along the shores of lake Victoria is an important supplementary source of food and income. A quarter of the population owns a television, 5% own a computer and 64% own a radio. Approximately 18% of persons aged 18 years and above are illiterate. Of the persons aged 6–12 years, 86% attend a primary school. Only 44% of persons aged 13–18 years attend a secondary school. The *Baganda,* who belong to the Bantu ethnic group, constitute most of the population in Mukono district. The Baganda inhabit the central and largest region of Uganda, and constitute the largest ethnic group in the country, accounting for 17% of the Ugandan population [21].

We conducted a descriptive qualitative study alongside a randomized controlled trial [22]. The randomised controlled trial was designed to assess the effectiveness of chlorhexidine use for umbilical cord care in the prevention of severe illnesses among newborns [23]. This formative study had two components: the first, reported in this paper, explored meanings and practices related to the umbilical cord; the other which investigated the acceptability of chlorhexidine for umbilical cord care, has been published elsewhere [24].

A total of 22 in-depth interviews exploring umbilical cord care perceptions and practices among ten mothers, four health workers, five traditional birth attendants (TBAs), and three fathers were conducted. The study participant characteristics are listed in Table 1 below. Participants were selected purposefully, mainly looking for participants with rich experiences in umbilical cord care and/or key decision makers in matters related to newborn care. None of the approached participants declined to participate.

**Table 1:** Study participant demographic characteristics

	Average (Av.) age	Av. education level	Av. No* of children
Young mothers	24	Secondary	2
Older mothers	52	Primary	6
TBAs	55	Primary	7
Health workers	41	Tertiary	_

Our gatekeeper, a TBA in the area, nominated mothers who had recently given birth. TBAs were also interviewed because they were maternal and neonatal health care providers in the community. Interviews were conducted face to face at calm and private settings away from distractions. Participants were recruited until the point of saturation [25] when no new themes emerged. Three focus group discussions (FGDs) were also conducted. Two were conducted at the health centre and involved young mothers recruited when they came to the health centre for postnatal follow-ups or immunization. The third FGD involved elderly women recruited through the gatekeeper (TBA) and was conducted at her home.We developed separate interview guides for the interviews with the different categories of study participants and topic guides for the different FGDs. The guides were used flexibly and modified according to the preliminary findings and as need arose in the course of the study [25]. The interviews usually lasted between 20 to 80 min. The first author (D.M.) conducted most interviews in *Luganda*, the local language and a few in English. A moderator and one note taker led the focus group discussions (FGDs). Participants were briefed on the main purpose of the discussion and emphasis was placed on inter participant discussions and confidentiality [26]. The first author wrote field notes to document impressions while in the field and our own experiences, beliefs, motives and assumptions. In addition to the IDI’s and FGDs, we used video recording to provide detailed description of the preparation and roles of *kyogero*, a local herbal mixture.

All the interviews were audiotaped. A professional transcribed and translated the interview/FGDs conducted in *Luganda*. The first author, who was present during all the interviews and has a good command of *Luganda*, proofread the translated transcripts. Data analysis started in the field and was an iterative process guided by qualitative content analysis [27], which included immersion in the data, identifying meaning units, abstracting content of meaning units and summarizing the importance (Table 2) [28]. Words, sentences or paragraphs that relayed a similar message were grouped as meaning units, which were then condensed and labelled with a code. I aggregated similar codes to form categories. Categories were made to be mutually exclusive, whenever that was possible and to include all the information related to the content area being discussed. Categories were further analysed to form manifest sub themes and themes. Based on this first analysis we identified Mary Douglas’ theories to take us to the next step of analysis/interpretation. This helped us define the latent themes. We used Nvivo 11.0.0 (QRS International, Cambridge, MA) to organize the analysis process.

**Table 2:** Examples of meaning units, codes, categories and themes from qualitative content analysis of interviews about umbilical cord care meanings and practices

Meaning unit/quotes	Code	Category	Theme
*The umbilical cord is the life of the child because it’s where the child breathes. If it’s cut off before time, the child could die. (Grandmother FGD)*	Source of life	Inside the womb: “The umbilical cord is the centre of life”	
*They fear the cord because it doesn’t look like the normal skin, and there is some little blood in the beginning, and as it shrinks it forms a certain discharge, which makes them think it’s painful for the baby. (Health worker IDI)*	Cord looks weird Fear of cord Cord is Painful	On the surface of the baby’s body: The cord as a source of tension	“The cord is the child” (Deduced theme/content area)
*There is even a saying that protect your child like you would protect the umbilical cord, the saying applies to anything, for example your phone, they say protect your phone like you would protect the umbilical cord, they say this because the cord has the function of proving that the child belongs to his clan*	Proving child clan	After detachment: “The cord is the passport to the clan”	

We triangulated the data collection methods by using IDIs, FGDs and Film, which increased the perspectives and deepened the understanding of the meanings attached to the umbilical cord and to umbilical cord care [29]. IDI’s enabled us to obtain individual experiences and perceptions and the FGDs enabled us to get group and cultural norms. The film captured the preparation of *kyogero* and was used for reference regarding this process. The first author was a master student at the time of the study, trained in qualitative methodology. Considering his medical training and gender, we initially worried that the study participants would be shy and not be open about their ideas and practices [30]. Rather, we experienced that his status as a medical doctor fostered trust, and hence did not seem to interfere with the way they described their understanding and practices. He belongs to a different ethnic group and did not know much about the culture of the participants, but assumed that participants were practicing what was being advocated at the health facilities. Two co-authors read through the interviews, codes, categories and themes as a way of peer examination, which increased credibility [31]. We did ‘participant checking’ [29] by discussing the findings with two study participants and then incorporated their reflections and additional information.

## Results

The findings are organized into two major sections. The first section focuses on the meanings attached to the umbilical cord, and the second section describes how the cultural understanding of the umbilical cord is reflected in umbilical cord care and other newborn care practices.

## The cord and its meaning: “The cord is the child”

We found that the umbilical cord carried symbolic meanings that extended beyond the health of the child and the newborn period. The way it was treated and kept reflected parental care and responsibility and was understood to be important for the future wellbeing of the child. One study participant explained the significance attributed to the cord by stating ‘the cord is the child’. We will attempt to uncover what this concept involved and to describe the various meanings associated with the cord. Using Mary Douglas’ conceptualisation of the body and its boundaries, and her elaboration of how matter changes meaning when it passes boundaries, we explore how the meaning of the cord changes as it transcends from inside the womb to the outside, and from the surface of the baby’s body until it falls off.

### Inside the womb: ‘The umbilical cord is the centre of life’

The participants appreciated the umbilical cord as a vital organ necessary for child growth in the uterus. Well in line with biomedical thinking, the umbilical cord was seen as a source of nourishment to the baby and was associated with life and death. A TBA told us: *We see this umbilical cord as the center of life (TBA IDI).* Explaining further, a participant in a FGD told us: *The umbilical cord is the life of the child because it’s where the child breathes. If it’s cut off before time, the child could die. (Grandmother FGD).*

Below, we shall see how the meanings/understandings of the umbilical cord change as the baby passes through the birth canal and is detached from the mother.

### On the surface of the baby’s body: the cord as a source of tension

After the baby is born, the umbilical cord is associated with anxiety and even fear. One participant told us:

*The umbilical cord looks like meat (TBA IDI)* and another said: *Mothers fear the umbilical cord because it looks like an intestine (Health worker IDI).* A health worker explaining the fear stated: *They fear the cord because it doesn’t look like the normal skin, and there is some little blood in the beginning, and as it shrinks it forms a certain discharge which makes them think it’s painful for the baby. (Health worker IDI)*

The discomfort and fear was also related to the perception that the umbilical cord was a vulnerable point on the child’s body and a point of weakness. Through the umbilical cord, various illnesses could enter the body of the child. These illnesses could be caused by natural agents as well as by social agents or strained social relationships. Diseases like tetanus and *Etumbizi* seemed to be explained in terms of natural agents while *Busobe* seemed to be caused by social conflict. *Etumbizi* was described as a disease caused by air entering the newborn, resulting in foaming around the mouth and subsequently death. *Etumbizi* could also result from negligence in covering the baby well. Describing *Etumbizi,* young mothers in an FGD told us: *That air can enter through the umbilical cord and affect the baby if you don’t cover him very well (FGD young mothers). Busobe* on the other hand was described as a disease caused by an adulterous father coming in contact with a newborn that still has an intact umbilical cord and causing convulsions. This was perceived as a very serious illness, with very few known effective cures and, in most cases, leading to death. A TBA described a case to us:

I saw it happen. Mrs. K’s child (not real name) almost died. The man came from somewhere and touched the baby and the baby started convulsing, its skin started turning yellow and it started producing foam from the mouth. (TBA IDI)

The child was seen as vulnerable to these illnesses for as long as the cord was still attached to the body of the baby and a mother illustrated this by saying: *Before a child’s umbilical cord is closed, I don’t count it as my child because the child could die any time (FGD young mothers).* As a result, there was a desire to have the umbilical cord detach as fast as possible to reduce this period of anxiety and uncertainty as a health worker informed us:

The cord is a delicate part on a baby and when it is still there, it gives you tension. So, it has to go off so that someone can be free. (IDI HW)

The smell of the umbilical cord before detachment also contributed to the anxiety associated with the umbilical cord:

The more it stays the more it smells. So, if you don’t keep it clean it smells a lot and even attracts houseflies. (TBA IDI)

During the period when the umbilical cord was on the surface of the baby, there was restricted access to the newborn to protect it from various dangers.

When I was growing up, my grandparent told us that the baby is not taken out of the house before the cord has broken off and you should not cross-junctions with the baby. They also used to tell us not to give the baby to people because you don’t know where they are coming from and don’t know what they have been doing. (TBA IDI)

### After detachment: “The cord is the passport to the clan”

After the umbilical cord fell off the newborn, it took on an ambiguous status. On one hand, it was seen as a treasure that should be kept safely as a TBA told us:

There is even a saying that protect your child like you would protect the umbilical cord, the saying applies to anything, for example your phone, they say protect your phone like you would protect the umbilical cord, they say this because the cord has the function of proving that the child belongs to his clan. (TBA IDI)

The umbilical cord was seen as a sign of belonging. As was further explained to us: *it’s [the cord is] the passport that allows you into the clan*. *(Mother FGD young women)* In the traditional practice known as *kwalula*, the cord was used to test if the child truly belonged to the father, and hence to the paternal clan.

The *kwalula* ceremony was usually performed at the husbands’ home and would either lead to jubilations if the traditional paternity test was positive or a rebuke and demand to return the child to the true father if it turned out negative. One participant explained the *kwalula* ceremony:

They get a basket lined with cow dung and put water in it, they smear ghee on the cord and then they put it in the basket containing water. If the cord sinks to the bottom of the basket then we rejoice and say that it is our child but if it comes to the top then we say the child is not ours. Even if you give birth to twenty children, you have to keep the cords from the first child to the last. (TBA IDI)

The *kwalula* ceremony was seen as an alternative to modern day DNA testing. The older mothers preferred *kwalul*a to DNA testing, as they perceived it as cheap, genuine and impossible to bribe, while the younger mothers preferred the accuracy offered by the current DNA testing. Women were expected to store the umbilical cord remnant very safely away from predators like rats. A misplaced cord remnant could be a source of unease as a young mother told us: *I am worried. I keep searching for it all the time, because if someone picks it, he can do something bad to my child. (Mother IDI).*

But a misplaced cord remnant could also be used to harm the mother as one of our study participants explained: *someone can perform witchcraft on the umbilical cord and the mother fails to conceive again. (Mother IDI).*At the same time safe handling of the cord remnant was seen as the responsibility of the mother and carelessness could harm the baby for instance by the cord getting into contact with a male child’s genitals: *The moment a woman leaves the cord to fall on the baby’s penis, the child becomes impotent. It is because the mother was careless. (TBA IDI).*

## Cord care practices

### Cutting the umbilical cord

Study participants were aware of the importance of hygiene and cleanliness when cutting the umbilical cord. Mothers who delivered from health centres reported the use of brand-new razor blades or surgical blades, usually part of delivery kits, during the cutting of their baby’s umbilical cord. For the deliveries that were conducted by the TBAs, effort was also made to ensure that the substances used to cut the umbilical cord were sterile as one TBA informed us:

Ever since we started, we have been using razor blades but if you are to use a razor blade, we usually have new razor blades, you get it when it is new. Now we mostly use scissors. But you also boil them; we do not use them before sterilizing. We do have stoves and local fireplaces for the sterilization process. You use usual water put the scissor in a pan, cover then boil like you boil something to eat then remove it and put on a clean plate to cool off so that it doesn’t burn you when you are using it. We do this to prevent germs from infecting the baby. (TBA IDI)

In the past, substances like elephant grass and bamboo were used to cut the umbilical cord but this was now seen as an old tradition that was no longer practiced in this society as a TBA informed us: *They would get a bamboo stick and use it to cut the umbilical cord. But now in our era we are using razor blades. (TBA IDI).*

### “A wound takes long to heal without treatment”

After cutting the umbilical cord, a number of substances were put on the umbilical cord and this was partly done to hasten drying and eventually to quicken umbilical cord fall off as a mother explained:

A wound without treatment takes long to heal but a wound on which you have applied medicine heals very fast. So if you have medicine that you apply, the cord goes off very fast. (Mother IDI)

Substances applied to the cord stump included: a herbal mixture locally known as *kyogero,* powder, banana (plantain) powder, ash, soap, normal saline, tealeaves, ghee, *kiyondo (a local herb) and dung (lizard and cow).*

There were also other reasons why substances were applied on the umbilical cord:

We do get surgical spirit from the hospital then you get cotton, dip it in the spirit, and then clean the umbilical cord. I think it is also to prevent it from rotting, smelling and to make it dry (TBA IDI)

There was also an awareness of the bio medical role of some substances applied on the umbilical cord in prevention of infections as a TBA told us:

We do get surgical spirit from the hospital then you get cotton dip it in the spirit, then clean the umbilical cord. I think it is also to prevent it from rotting, smelling and to make it dry (TBA IDI)

Substances were also applied after the umbilical cord fell off, mainly to prevent umbilical colic:

Most people use the kiyondo after the cord has fallen off because after the cord falls off, it leaves something like a wound. So our elders say that the “kiyondo” prevents the baby from abdominal colic and over crying. (Mother IDI)

Other substances applied on the umbilical cord to prevent colic included tomato extract, saliva and mushroom *(obutiko obubaala)* as a mother explained to us:

After the cord had fallen off they told me to put mushrooms. They said that they put the mushrooms in a banana leaf then steam it, after bathing the baby you squeeze water out of the mushrooms then put drops on the umbilical cord. (Mother IDI)

Healthcare workers often gave varying instructions of umbilical cord care:

One tells you use, the other tells you don’t use. And we in the community get confused. (TBA IDI)

### “Our newly born child should not be bathed in plain water”

The bathing of a newborn was a topic that was brought up by most participants and was indirectly associated with the umbilical cord. Bathing was important because the umbilical cord was cleaned during bathing. Newborns were usually bathed in a herbal solution called *kyogero*: *A child from Buganda shouldn’t be bathed in plain water. (FGD older women).*

*Kyogero* is a mixture of several herbs, boiled together and the resulting solution used to bath the newborn, and sometimes dropped in the mouth and on the umbilical cord. *Kyogero* mixture is often re-used because it is very cumbersome and expensive to prepare. Children were bathed in *kyogero* so that they would be blessed. These blessings could manifest in various ways: having a healthy childhood, good financial and marital fortune later in life and having a good personality.

Now for example a girl getting married to a rich man who also has good manners. Have you ever sat down and said so and so’s children are all married to rich men or they all have jobs. So that is one of the benefits of the kyogero according to our grandparents (TBA IDI)

*Kyogero* was also popular among the midwives as the head of the maternity ward told us:

They believe in it so much including the midwives who are trained. So they feel those are drugs. They call them herbal medicines, which help the baby to get a clear skin like I said good luck and all those sorts of things. (Health worker IDI)

Many of the younger mothers did not know how to prepare *kyogero* and left this role to their older female relatives. Most of the herbs are no longer available in urban areas and have to be obtained from rural areas. When asked what they use if they don’t have access to kyogero participants told us:

Most people use soap with Vaseline and powder for example Johnson or they use commercially prepared kyogero soap if they don’t have the kyogero, (FGD young mothers)

There were also a few participants who said they had never used or heard about *kyogero:*

At our home I have seen our mothers delivering children but they don’t use kyogero and yet their children grow up well. Because of that, I have never bathed my son in kyogero. (FGD young mothers)

## Discussion

The umbilical cord took up different meanings as it transitioned from being inside the mother’s body to detaching off the child’s body. Inside the body, the cord, in accordance with medical thinking, was seen as vital for the nutrition of the child. On the surface of the baby’s body, the cord was a source of anxiety and fear, hence the use of substances to quicken its detachment. After detachment, the cord took on an ambiguous nature; on one hand it was a very important item used to confirm the paternity of a child, on another hand it could result in impotence if it touched the genitals of a boy. The way it was treated and kept reflected parental care and responsibility and was important for the future wellbeing of the child. Using Mary Douglas’ conception of body boundaries and of how matter changes meaning as it passes boundaries, we try to understand the meaning of the cord and of the practices related to it.

The appreciation of the importance of the umbilical cord inside the uterus has also been reported in a similar study in Zambia [2]. However after birth when the umbilical cord was cut and tied and it lingered on the surface of the newborn body, the umbilical cord became a source of fear, dread and uncertainty. Mary Douglas’ boundary perspectives assist us to understand the fear and anxiety related to the umbilical cord. She argues that substances which defy boundaries, or threaten the proper separation offered by boundaries are treated as dangerous [20] due to their ambiguity, and one way of dealing with this uncertainty is the separation of such substances from the body [20]. When the umbilical cord is on the surface of the body, it breaks the boundary that should be offered by the skin, by serving as an alternative connection between the outside and the inside of the body and this could explain the anxiety. This perception of anxiety is in line with the anthropological concept of liminality. Liminality is a transitional period where the status of the subject is unclear and while it has left one category, it has not yet entered another [32]. A commonly cited example is a young pregnant woman who is transitioning from being a girl to becoming a mother. The period of liminality elicits anxiety and unease. It commonly results in protective measures, but also in the desire for this period to end as fast as possible. In this case the newborn is seen as no longer a foetus, but not yet recognised as a baby as illustrated by the mother who explained that she could not count the baby as a child until the cord had fallen off. The umbilical cord fall off marks the end of this liminal phase.

This perception of fear and anxiety could also be attributed to the way the umbilical cord physically appeared, with some participants stating that it ‘looked like an intestine’ an organ expected in the interior of the body. Newborns with an umbilical cord on their surface were seen as very vulnerable to both physical and spiritual attacks, a finding reported by other authors [6, 33]. This discomfort and unease with the exteriorized umbilical cord may help to explain many umbilical cord care practices. Underlying most umbilical practices was the desire to have the umbilical cord separate as fast as possible, in order to reduce the period of anxiety but also to ensure wholeness of the body through the closure of the umbilical cord. The desire of mothers to have the umbilical cord separate quickly has been reported in a number of studies from Uganda, Zambia, Ethiopia and other parts of Africa [2, 6, 34–37]. The umbilical cord also produced secretions which mothers were uncomfortable with. Powdered substances like plantain ash, normal ash, lizard excreta, and baby powder were perceived to absorb these secretions and quicken the speed of drying of the umbilical cord. Other liquid substances like the herbal mixture *kyogero* and surgical spirit are also perceived to quicken the umbilical cord drying and separation. The concept of applying substances on the umbilical cord to quicken its detachment has been reported in studies from other African settings [2, 35, 36, 38]. When we asked some participants about what they thought was the single most important characteristic of a substance designed for umbilical cord care, most of them stated that the substance should quicken umbilical cord separation. To further illustrate how desire for umbilical cord separation influenced umbilical cord care practices, unfamiliar persons and extended family were discouraged from carrying newborns until the umbilical cord separated, a way of protecting the newborn from ailments. Nalwadda and colleagues [39] found similar concepts in Eastern Uganda where children ceased to be called newborns as soon as the umbilical cord separated. The issue of umbilical cord separation is therefore very important when discussing umbilical cord care with mothers of newborns.

The meaning and perception of the umbilical cord changed when the umbilical cord had detached from the baby. In the detached form, the umbilical cord took on a mixed identity. The umbilical cord was generally highly regarded, and well kept, as it was used to perform traditional paternity rituals that assessed whether the child truly belonged to the father. This symbolism surrounding the umbilical cord could be harnessed in future endeavours to justify storage of umbilical stem cells [40]. On the other hand, the detached umbilical cord was thought to cause impotence if it touched the genitalia of the male child. Similar perceptions have been document in Zambia [2]. This change in perception and meanings resonates with Douglas’ theory about dirt being a matter out of place (22). As long as the umbilical cord stump stays within its symbolic borders, it is harmless, or even appreciated. The male genitals seem to constitute a symbolic border for the cord stump, and if the stump is to cross that border, we can understand it as being “matter out of place” and thereby dirty, or even dangerous [17].

One way of dealing with the perceived vulnerabilities associated with the umbilical cord, and beyond, was bathing the newborn in the herbal solution *kyogero.* From a biomedical perspective, whereas we fear the possible contamination that *kyogero* might cause, what is of more concern is the reuse of *kyogero*. *Kyogero* is kept for about one to two weeks and warmed to body temperature prior to bathing the baby, something that could favour bacterial growth and cause infections in the newborn. In Risk and Blame, Mary Douglas argues that risk assessment does not rest on individuals’ cognitive abilities but on socially predetermined assessments, which are politically and morally built. Douglas argues that a refusal to take sound hygienic advice should therefore not be attributed to weakness of understanding but on preference. With this understanding, policies that are targeted towards umbilical cord care should acknowledge the practice of herbal washing of newborns, and find ways of co-existing with it. This could be by encouraging newborns to delay washing the baby until the umbilical cord separates.

Despite the reduction in availability and popularity of herbs such as *kyogero* among the younger urban dwelling mothers, the herbs are still commonly used. This is, at least partly, because it is older female relatives who take care of newborns in the first days after birth. This demonstrates the importance of involving such persons in interventions promoting safe umbilical cord care.

Because the study has investigated practices that have also been documented in other areas of sub-Saharan Africa, we argue that the lessons learnt are transferable to similar socio-economic contexts. Hence, the study has implications to the scale up of recommended newborn care practices beyond Uganda. Prior efforts to scale up such recommended practices have been unsuccessful and this could be related to the poor understanding of the cultural meanings surrounding newborn care [34]. The insights obtained from understanding the symbolic value of the umbilical cord and how this relates to umbilical cord care can contribute to the development of culturally acceptable guidelines with greater possibility of being adopted by members of the community. The fear and danger associated with the cord is paralleled with the risk of infections in biomedical thinking. What differs is the reasoning about cause and the practices to prevent illness. Interventions designed to promote safer cord care should leverage on its symbolic value and address issues related to illness causation, and practices springing out of these beliefs.

### Study limitations

In studying a community practice like umbilical cord care, participant observation would have been an appropriate research approach, but due to the sacredness that was attached to the birth process and the early neonatal period we were unable to conduct observations. We also did not empirically explore the association of the umbilical cord with other newborn care practices like skin-to-skin care, and home visits; practices that could possibly be affected by beliefs surrounding the umbilical cord.

## Conclusions

The umbilical cord carried symbolic meanings, which extended beyond the newborn and the newborn period, and in turn influenced the various practices of umbilical cord care. Unless treated with care, the umbilical cord was seen to cause immediate danger to the survival as well as the long-term wellbeing of the child. Public health interventions addressing newborn care should recognize the important position of the umbilical cord in the local idea system and capitalize on the perceptions of risk associated with the cord when introducing new evidence based newborn care practices.

## Data Availability

Data generated and analyzed during this study are not publicly available due to potential breech of confidentiality, but are available from the corresponding author on reasonable request. The data obtained is in the form of audio recordings and verbatim transcripts, which are very difficult to remove all personal identifiers.

## Abbreviations

CHX: Chlorhexidine
FGD: Focus Group Discussion
IDI: In Depth Interview
RCT: Randomized Controlled Trial
TBA: Traditional Birth Attendant
WHO: World Health Organization
